# Health-related quality of life and costs of switching originator infliximab to biosimilar one in treatment of inflammatory bowel disease

**DOI:** 10.1097/MD.0000000000018723

**Published:** 2020-01-10

**Authors:** Saara Huoponen, Anja Eberl, Pirjo Räsänen, Risto P. Roine, Taina Sipponen, Perttu Arkkila, Marja Blom

**Affiliations:** a University of Helsinki, Helsinki; b ESiOR Oy, Kuopio; c Helsinki University Hospital, Helsinki; d University of Eastern Finland, Kuopio, Finland.

**Keywords:** biosimilar, Crohn disease, health-related quality of life, inflammatory bowel disease, infliximab, ulcerative colitis

## Abstract

Effectiveness, efficacy and safety of biosimilar infliximab (CT-P13) in inflammatory bowel disease (IBD) patients has been shown in previous studies. Limited data exist on health-related quality of life (HRQoL) of switching originator to biosimilar infliximab (IFX) in IBD patients. The objective of this study was to evaluate impact of switching originator to biosimilar IFX on HRQoL, disease activity, and health care costs in IBD maintenance treatment.

In this single-center prospective observational study, all IBD patients receiving maintenance IFX therapy were switched to biosimilar IFX. HRQoL was measured using the generic 15D health-related quality of life instrument (15D) utility measurement and the disease-specific Inflammatory Bowel Disease Questionnaire (IBDQ). Crohn Disease Activity Index (CDAI) or Partial Mayo Score (pMayo), and fecal calprotectin (FC) served for evaluation of disease activity. Data were collected at time of switching and 3 and 12 months after switching. Patients’ characteristics, clinical background information and costs were collected from patient records and the hospital's electronic database.

Fifty-four patients were included in the analysis. No statistically significant changes were observed in 15D, CDAI, pMayo, and FC during 1-year follow-up. IBDQ scores were higher (*P* = .018) in Crohn disease 3 months after switching than at time of switching. Costs of biosimilar IFX were one-third of costs of originator one. Total costs related to secondary health care (excluding costs of IFX), were similar before and after the onset of biosimilar IFX.

HRQoL and disease activity were after switching from originator to biosimilar IFX comparable, but the costs of biosimilar IFX were only one-third of those of the originator one.

## 1 Introduction

Inflammatory bowel diseases (IBD), including Crohn disease (CD), ulcerative colitis (UC), and IBD unclassified (IBD-u), are conditions characterized by chronic inflammation of the gastrointestinal tract. The worldwide incidence of IBD has increased since the latter part of 20th century affecting mainly young adults and causing an increasing economic burden.^[1,2]^ During the last two decades, the use of the biological drugs has increased significantly. Biologics have been shown to be effective in inducing and maintaining remission in IBD.^[3,4]^ However, they are significantly more expensive than conventional drugs. Therefore, interest has grown in biosimilars that are comparable to the originator product in terms of efficacy and safety. In June 2013, the biosimilar infliximab (IFX), CT-P13, was accepted by the European Medicines Agency (EMA) for all indications of the originator product.^[5]^ Studies demonstrating efficacy of biosimilar IFX were conducted in ankylosing spondylitis and rheumatoid arthritis, and these results were extrapolated for IBD, causing concern in IBD societies.^[6–8]^ The effectiveness, efficacy and safety of biosimilar IFX in IBD patients has been shown in previous studies.^[9–15]^ The objective of this study was to evaluate HRQoL, disease activity, and health care costs before and after switching originator to biosimilar IFX in the maintenance treatment of Finnish IBD patients.

## 2 Methods

### 2.1 Study design

**Figure 1:**
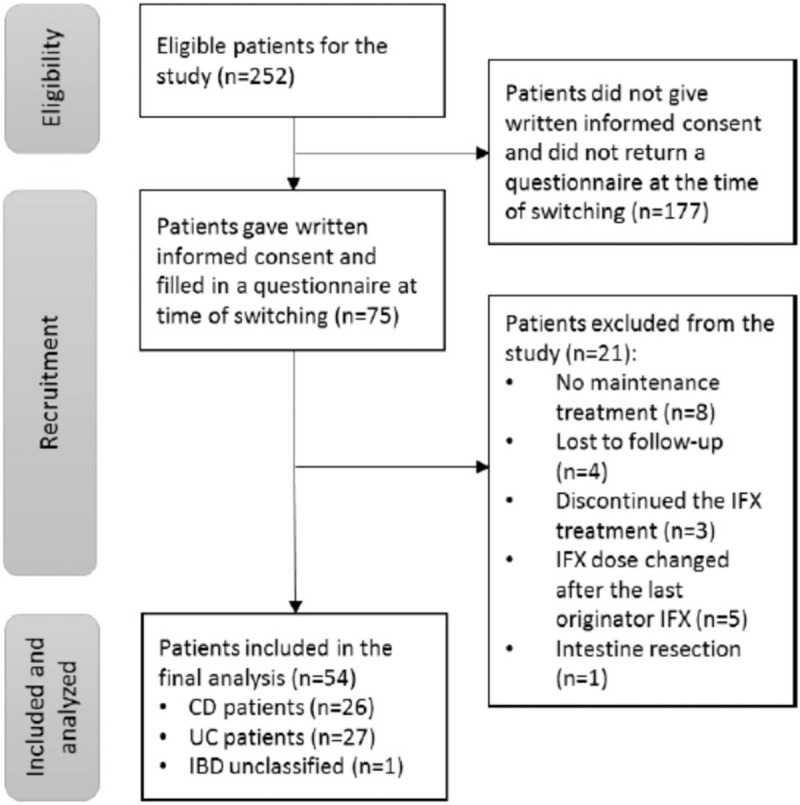
Flowchart of the patients included in the study. CD = Crohn disease, IBD = inflammatory bowel disease, IFX = infliximab, UC = ulcerative colitis.

In this investigator-initiated, prospective, observational, single-center study, all adult IBD patients (≥18 years) receiving maintenance IFX (Remicade^TM^, Janssen Biotech, Inc/ Schering-Plough, EU) treatment at Helsinki University Hospital were switched to biosimilar IFX (Remsima^TM^, Celltrion Pharm, Inc**.,** South Korea) in the beginning of 2016. Before the administration of the first biosimilar IFX in the day care unit, all IBD patients with IFX treatment were asked to participate in the study (Fig. 1). Whether patients chose to participate in the study or not, all adult IBD patients (≥18 years) receiving maintenance IFX therapy were switched to a biosimilar one, and they received all the services they usually did. At the time of administration of the first biosimilar IFX, IBD patients were asked to complete a questionnaire concerning HRQoL and disease activity. Patients who had returned the first questionnaire and the informed consent form were asked to answer follow-up questionnaires at 3 and 12 months after switching. One reminder was sent to patients who had not returned the follow-up questionnaires.

The generic HRQoL measure used in this study was 15D health-related quality of life instrument (15D), which is 15-dimensional, standardized, and self-administered instrument that can be used both as a profile and as a single index score measure covering the most important dimensions of health including mobility, vision, hearing, breathing, sleeping, eating, speech (communication), excretion, usual activities, mental function, discomfort and symptoms, depression, distress, vitality, and sexual activity.^[16,17]^ Each dimension is divided into five levels from no problems to extreme problems. The single-index 15D score, representing the overall HRQoL on a scale of 0–1 (0 = being dead, 1 = no problems on any dimensions) calculated from the health state descriptive system by using a set of population-based preference or utility weights. A change of approximately ±0.015 in the total 15D score is considered clinically or practically important because people can usually sense such a magnitude of change.^[18]^ If patient did not answer all the questions of the 15D, one to three missing responses were predicted with linear regression analysis using other dimensions, age, and sex as independent variables.^[19]^

Disease-specific HRQoL information was collected with the IBDQ. The IBDQ is a widely used, standardized measure for assessment of HRQoL in patients with IBD. The IBDQ includes 32 items, which are divided into four subscales: gastrointestinal symptoms (10 items), systemic symptoms (5 items), emotional function (12 items) and social function (5 items) and each item is scored on a 7-point scale, ranging from 1 to 7 (worst to best of health).^[20]^ The total IBDQ scores may range from 32 to 224, with higher scores representing better health. The scores of patients in clinical remission usually are 170 points or more.^[21]^ A clinically meaningful improvement is an increase ≥16 points in the total IBDQ score in CD, and a mean decrease in relapse is about 32 points.^[21,22]^ One unanswered item of IBDQ subscale was replaced by the mean of the items of the respective subscale. The IBDQ questionnaire was excluded from further analyses if more than one item of subscale was missing. The IBDQ was used under license from McMaster University, Hamilton, Canada.

Clinical disease activity assessment in CD was based on the CD Activity Index (CDAI).^[23]^ CDAI less than 150 indicates remission, and scores 150 to 219 mildly, 220 to 450 moderately, and >450 severely active disease. In UC and IBD-u, clinical disease activity assessment was based on the partial Mayo Score (pMayo). The numerical results provide a score ranging from 0 to 9 that represents an estimate of clinical activity of UC, and clinical remission was defined as pMayo < 2.^[24]^ Fecal calprotectin (FC) served for objective measurement of disease activity and was measured by a quantitative enzyme immunoassay (PhiCal Test, Calpro AS, Oslo, Norway). The FC values quoted as normal were <100 μg/g of stool.^[25]^ The patients’ clinical background information regarding the disease state and the treatments given were collected from the hospital records.

The study was conducted from the healthcare provider perspective. The costs of production of the services were obtained from the hospital accounting records of the Helsinki University Hospital (HUS), where all costs of specialized health care of individual patients were stored on a routine basis.^[26–29]^ The total cost data covered all costs related to the secondary health care provider (intervention, ward, ambulatory visits, laboratory, radiology, pathology, outpatient visits). Regardless of the diagnosis, all costs of specialized health care of IBD patients were analyzed. No productivity costs or outpatient drug costs were included. Costs of IFX were calculated based on the hospital records. Discounting was not considered in the study as the data were based only on years 2015 and 2016. All costs were converted to 2017 euros using the health care price index of Statistics Finland.^[30]^ The primary outcomes of the study were the changes in HRQoL, disease activity, and costs during the follow-up up.

### 2.2 Statistical analyses

The statistical significance of the difference in mean 15D scores during follow-up was tested with paired samples *t* test. As the 15D variables were not normally distributed and the patient size was relatively small, the Wilcoxon Signed Rank test as non-parametric test was also applied. Aside from minor differences in the level of statistical significance, the results of parametric and non-parametric tests were similar. Therefore, only results of parametric tests are reported for the 15D. The Wilcoxon Signed Rank test was used to test the statistical significance between variables in IBDQ, CDAI, pMayo, FC, health service use and costs during the follow-up. The Mann-Whitney *U* Test was used to examine differences between patients included and excluded from the study. The results are given as mean and standard deviation (SD) or as median and interquartile range (IQR). Values less than 0.05 were considered statistically significant. Statistical analyses were performed for CD and UC patients, and patients with IBD-u were included in the UC group. Subgroup analyses were conducted for patients in remission at the time of switching. The data were analyzed using IBM SPSS Statistics 24 (SPSS, Inc.).

### 2.3 Ethical considerations

Ethical approval was granted by the Ethics Committee of Medicine of HUS (32/13/03/01/2016). The research permit was given by the HUS (HUS-170-2016-2 and HUS-333-2019-23). All participants signed informed consent form.

## 3 Results

### 3.1 Patients

Of the 252 eligible IBD patients, 75 were willing to participate and returned their informed consent and questionnaire at the time of switching (Fig. 1). A total of 21 patients were excluded from the study, and, consequently, 54 patients were included in the final analysis. Of these 54 patients, 48 (88.9%) and 43 (79.6%) replied to the questionnaire at 3 and 12 months after the switching. The characteristics of the patients included in the study are presented in the Table 1. Patient characteristics were similar between patients included (n = 54) and excluded (n = 21) from the final analysis, except in the duration of IFX treatment (*P* = .017) and the location of CD according to the Montreal classification (*P* = .007) in CD. Of the patients who discontinued the IFX treatment (n = 3), 1 patient switched to another biologic treatment, 1 patient achieved remission, and one patient discontinued IFX treatment based on IFX trough levels. Two patients, who were lost to follow-up, had moved out of the hospital district.

**Table 1:**
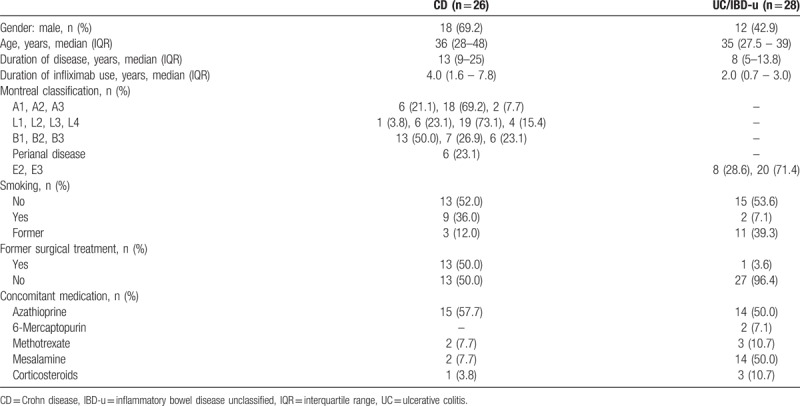
Patient characteristics at the time of switching.

### 3.2 Health-related quality of life

**Figure 2:**
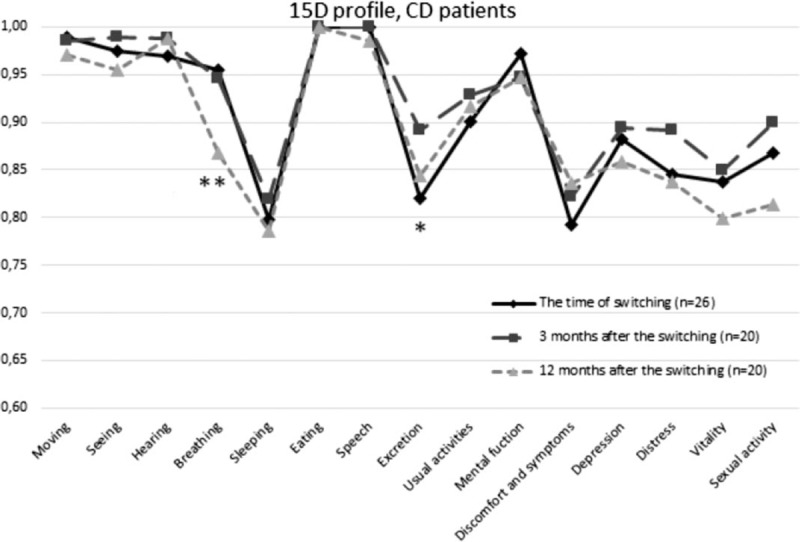
The 15D profile in Crohn disease patients. ^∗^statistically significant difference (*P* < .05) from the time of switching to 3 months after switching. ^∗∗^statistically significant difference (*P* < .05) from the time of switching to 12 months after switching. Paired samples *t* test was used to test statistical significance.

**Figure 3:**
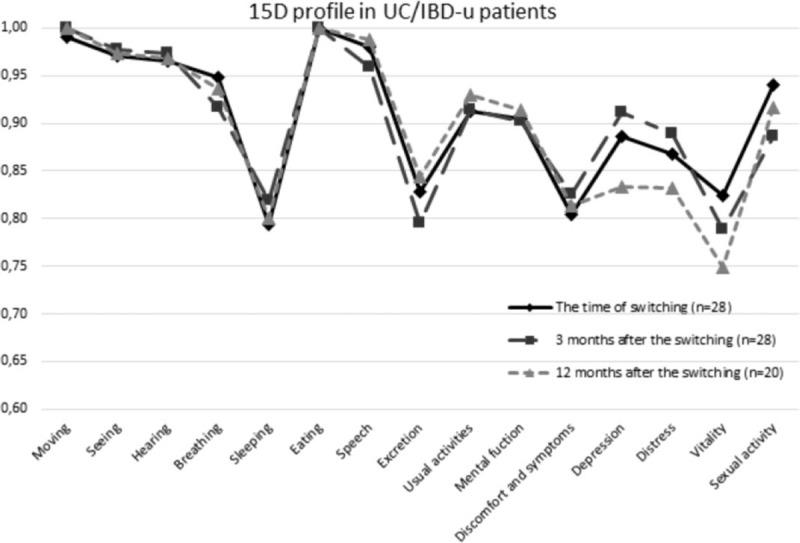
The 15D profile in ulcerative colitis and inflammatory bowel disease unclassified patients. Paired samples *t* test was used to test statistical significance. IBD-u = IBD-unclassified, UC = ulcerative colitis.

At the time of switching and at 3 and 12 months after it, the 15D scores in the whole study group ranged from 0.745 to 1, from 0.687 to 1, and from 0.700 to 1, respectively. At the same points of measurement, full health (the 15D score = 1) was reported by 12%, 20%, and 15% of CD patients, respectively, and by 7%, 7%, and 17% of UC patients, respectively. Compared to the time of switching, the mean 15D scores in CD (*P* = .310 and *P* = .129) and in UC patients (*P* = .470 and *P* = .319) did not differ at 3 and 12 months, respectively (Table 2). A clinically important difference was not observed in UC, whereas in CD patients the change in the total 15D scores between 3 and 12 months was clinically important. The proportion of patients that experienced at least a minimum clinically important change in HRQoL 12 months after the switching was 40% for improvement and 31% for deterioration in the whole study group (Table 3). Regarding the different dimensions, statistically significant difference was observed in excretion (*P* = .042) and in breathing (*P* = .021) in CD as compared to the time of switching, whereas in UC no statistically significant differences (all *P* > .05) on any dimension were not observed (Figs. 2 and 3).

**Table 2:**
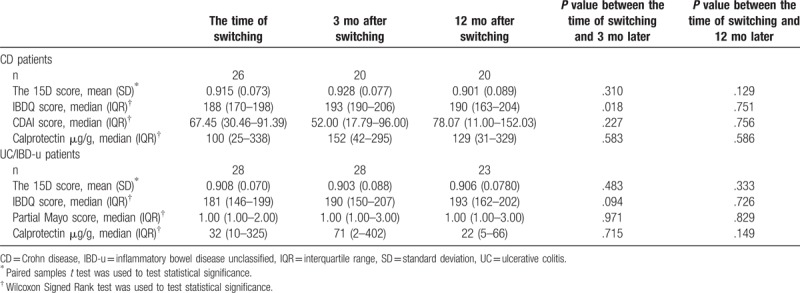
Health-related quality of life and disease activity.

**Table 3:**
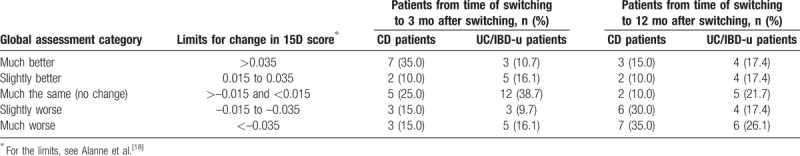
Classification of the changes in 15D scores from the time of switching to 3 and 12 months into global assessment scale categories and the distribution of the patients into these categories.

At the time of switching and 3 and 12 months later, IBDQ score ≥170 (considered remission) was reported by 73%, 85%, and 70% of CD patients, respectively, and by 64%, 69%, and 70% of UC patients, respectively. Statistically significant improvement (*P* = .018) was observed in IBDQ scores at 3 months after switching in CD (Table 2). Compared to the time of switching, statistically significant difference was observed neither in CD (*P* = .088 and *P* = .932) nor in UC patients (*P* = .117 and .586) at 3 and 12 months, respectively, when patients in remission at the time of switching were only considered. The percentages of patients who met an IBDQ change ≥16 (considered clinically meaningful improvement) was 5% (n = 1) in CD and 17% (n = 4) in UC 12 months after switching. The total IBDQ score decreased by more than 32 points (considered relapse) in one UC patient 12 months after switching.

### 3.3 Disease activity

At the time of switching and at 3 and 12 months later, a CDAI less than 150 (considered remission) was reported by 92%, 89%, and 63% of CD patients, respectively. pMayo score of <2 (considered remission) was reported by 63%, 63%, and 76% of UC patients, respectively. At respective points of measurement, median FC concentration was 82 (IQR 13–312), 83 (IQR 26–300), and 33 (IQR 10–186) in the whole study group. Compared to the time of switching, changes in the CDAI (*P* = .227 and *P* = .756), pMayo (*P* = .971 and *P* = .829), or FC concentration (CD: *P* = .583 and .586; UC: *P* = .715 and .149) showed no difference at 3 and 12 months, respectively (Table 2). When patients in remission at the time of switching were only considered, changes in the CDAI (*P* = .227 and *P* = .826) and pMayo (*P* = .317 and *P* = .157) were not significantly different at respective time points.

### 3.4 Health service use and costs

The number of IFX administration visits and IFX doses between 2015 (IFX originator used) and 2016 (IFX biosimilar used) showed neither in CD (*P* = .590 and *P* = .109) nor in UC (*P* = .372 and .225) a statistically significant difference (Table 4). Costs of IFX were significantly higher (*P* < .001) in 2015 than in 2016. Costs of IFX in 2016 were 35% of costs of IFX in 2015. The mean total costs covering all costs related to the secondary health care provider (intervention, ward, ambulatory visits, laboratory, radiology, pathology, outpatient visits, and excluding the costs of IFX) were 4405 € (SD 4132) per patient in the whole study group in 2015, while costs were 3830 € (SD 2573) in 2016. When patients who started IFX treatment in 2015 (4 CD patients and 10 UC/IBD-u patients) were excluded, the mean total costs were 3804€ (SD 3771) per patient in 2015 and 3886 € (SD 2597) per patient in 2016. Total costs related to secondary health care (excluding the costs of IFX) between 2016 and 2015 showed neither in the whole study group (*P* = .799) nor in patients who had started IFX treatment before 2015 a difference (*P* = .340).

**Table 4:**
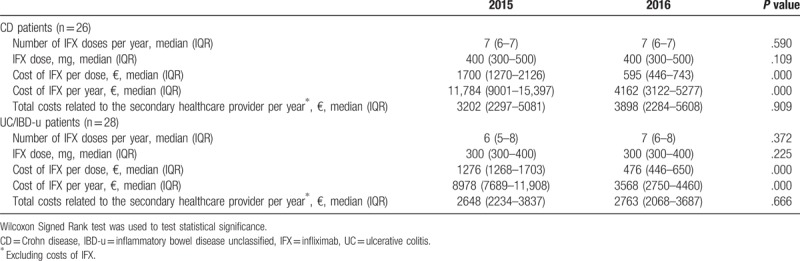
Infliximab doses and costs per patient in a specialized health care.

## 4 Discussion

The aim of this single-center observational study was to evaluate HRQoL, disease activity, and costs of switching originator to biosimilar one in the maintenance treatment of Finnish IBD patients. Based on this study, HRQoL measured by the generic 15D of biosimilar IFX was comparable to that during originator one in the 1-year study follow-up. Statistically significant improvement was observed in IBDQ scores between the time of switching and 3 months in CD. However, statistically significant difference was not observed when CD patients in remission at the time of switching were only considered. There were no changes in clinical or FC-measured disease activity after switching. The costs of biosimilar IFX were around one-third of the costs of originator one, whereas costs related to secondary health care (excluding the costs of IFX) were similar in 2015 (originator IFX used) and 2016 (biosimilar IFX used).

Consistent with our results, previous studies showed comparable disease activity between originator IFX and biosimilar one.^[9–14]^ Different from the previous studies, our study showed statistically significant improvement in IBDQ scores 3 months after the switching in CD as compared with the time of switching. However, a statistically significant difference was not anymore present at 12 months after the switching. Furthermore, the generic HRQoL measure, the 15D suggest that IFX biosimilar is comparable to originator one, and therefore, results of IBDQ should be interpreted with caution. It is unclear whether the change in IBDQ scores is due to the switching to IFX biosimilar or due to other patients’ characteristics or clinical background information.

The strength of this study is its prospective nature and that all adult IBD patients (≥18 years) receiving maintenance IFX therapy were systemically switched to biosimilar one whether they participated in the study or not. It is also notable, that study population consisted of IBD patients treated in a tertiary clinic, representing IBD patients most difficult to treat. Another strength of this study was its relatively long follow-up as both HRQoL and disease activity were measured at the time of switching and at 3 and 12 months later. Furthermore, both clinical and objective disease specific measures were used to evaluate disease activity. In this study, the mean 15D and IBDQ scores of IBD patients during 1-year follow-up were 0.901 to 0.928 and 175.93 to 190.94, respectively. These scores are higher than the mean 15D score of 0.874 and the mean IBDQ score of 163 in Finnish IBD patients reported by Haapamäki et al.^[31]^ Additionally, the mean 15D score was 0.868 for the Finnish IBD patients of the Health 2000 survey.^[32]^ Based on the NOR-SWITCH study, the mean IBDQ score in IBD was 187 to 190, which is comparable to the mean IBDQ score of this study.^[12]^ The 15D score has shown to be strongly related to the total IBDQ score and disease activity.^[31]^ According to the study by Haapamäki et al, the total 15D scores of IBD patients in clinical remission can be estimated to be about 0.89 or higher.^[31]^ In this study, the 15D score of 0.89 or higher was observed by 65%, 75%, and 60% of CD patients and by 57%, 68%, and 65% of UC patients at time of switching and 3 and 12 months later, respectively. This finding is in line with IBDQ, CDAI, and pMayo, as most IBD patients in this study were in remission.

This study showed that costs of biosimilar IFX were significantly lower than costs of originator one. The costs of production of the services (excluding the costs of IFX) were obtained from the hospital accounting records by HUS,^[26–29]^ where all costs of specialized health care of individual patients were analyzed regardless of the diagnosis. Therefore, costs of specialized health care were not IBD-related, but this study showed that IFX biosimilar had no impact on the costs related to specialized health care (excluding the costs of IFX). Adalimumab has also reached, and many other biologic drugs are approaching, patent expiry. Biosimilar drugs offer considerable cost reductions in health care, and in turn, biologic treatment may become available for a larger number of IBD patients.

Among the limitations of our study is a lack of a control group continuing originator IFX. Another limitation is a relatively small patient number. Although patient characteristics were similar between patients included (n = 54) and excluded (n = 21) from the final analysis, except for CD patients in the duration of IFX treatment (*P* = .017) and the location of CD according to the Montreal classification (*P* = .007), a weakness of this study is the unknown difference between non-participants (n = 177) and participants (n = 75). Those who did not take part in the study may have been unaware of their symptoms and their impact on HRQoL. It is also notable that patients were aware of the fact that they switched to biosimilar IFX which may have influenced the results. No productivity costs or outpatient drug costs were analyzed in this study. The status of working and number of sickness leave days were asked in the questionnaire but not analyzed in the study due to defective data.

In conclusion, this study suggested that in maintenance therapy of IBD biosimilar IFX was, in light of the 15D, CDAI, pMayo, and FC, comparable to originator one during 1-year study follow-up. The costs of biosimilar IFX were around one-third of the costs of originator one, whereas costs related to secondary health care were similar before and after the onset of biosimilar IFX.

## Author contributions

All authors contributed to the study conception and design, material preparation, data collection and analysis. The statistical analyses were performed by SH. The first draft of the manuscript was written by SH and all authors commented on previous versions of the manuscript. All authors have read and approved the final manuscript.

Saara Huoponen orcid: 0000-0001-7209-0963.
